# Actin-related proteins regulate the RSC chromatin remodeler by weakening intramolecular interactions of the Sth1 ATPase

**DOI:** 10.1038/s42003-017-0002-6

**Published:** 2018-01-22

**Authors:** Bengi Turegun, Richard W. Baker, Andres E. Leschziner, Roberto Dominguez

**Affiliations:** 1 Department of Physiology, Perelman School of Medicine, University of Pennsylvania, 728 Clinical Research Building, 415 Curie Boulevard, Philadelphia, PA 19104-6085 USA; 2 Department of Cellular and Molecular Medicine, School of Medicine, and Section of Molecular Biology, Division of Biological Sciences, UC San Diego, 9500 Gilman Drive, La Jolla, CA 92093 USA

## Abstract

The catalytic subunits of SWI/SNF-family and INO80-family chromatin remodelers bind actin and actin-related proteins (Arps) through an N-terminal helicase/SANT-associated (HSA) domain. Between the HSA and ATPase domains lies a conserved post-HSA (pHSA) domain. The HSA domain of Sth1, the catalytic subunit of the yeast SWI/SNF-family remodeler RSC, recruits the Rtt102-Arp7/9 heterotrimer. Rtt102-Arp7/9 regulates RSC function, but the mechanism is unclear. We show that the pHSA domain interacts directly with another conserved region of the catalytic subunit, protrusion-1. Rtt102-Arp7/9 binding to the HSA domain weakens this interaction and promotes the formation of stable, monodisperse complexes with DNA and nucleosomes. A crystal structure of Rtt102-Arp7/9 shows that ATP binds to Arp7 but not Arp9. However, Arp7 does not hydrolyze ATP. Together, the results suggest that Rtt102 and ATP stabilize a conformation of Arp7/9 that potentiates binding to the HSA domain, which releases intramolecular interactions within Sth1 and controls DNA and nucleosome binding.

## Abstract (web summary)

Bengi Turegun *et al.* report an interaction of the highly-conserved pHSA and P1 domains of Sth1, the catalytic subunit of the SWI/SNF-family chromatin remodeler RSC. This interaction is released when ATP-bound Rtt102-Arp7/9 binds to the HSA domain, modulating DNA and nucleosome binding by Sth.

## Introduction

Chromatin-remodeling complexes (remodelers) are large, multisubunit complexes that regulate gene expression and genome maintenance^1^. Remodeler malfunction is often linked to diseases, including cancer^2^ and cardiovascular disorders^3^. Remodelers belong to four families: CHD, ISWI, SWI/SNF, and INO80. While the subunit composition of these families varies, they all assemble around a catalytic subunit defined by the presence of a conserved helicase superfamily 2 (SF2) ATPase domain^4, 5^. The ATPase domain consists of two recA-like subdomains, with the nucleotide-binding and DNA-binding sites located at the interface between subdomains^6–8^. DNA-dependent ATP hydrolysis by the catalytic subunit propels changes in chromatin structure, such as histone octamer sliding, ejection, and histone subunit exchange^9–14^. These activities are regulated by auxiliary subunits recruited through family-specific domains N and C terminal to the central ATPase domain of the catalytic subunit.

Two families of remodelers, SWI/SNF and INO80, recruit as auxiliary subunits actin and actin-related proteins (Arps) through a region N terminal to the ATPase domain called the helicase/SANT-associated (HSA) domain^15^. Actin and Arps have been proposed to regulate the catalytic and nucleosome targeting and sliding activities of their host remodelers^15–22^. The HSA domain of Sth1, the catalytic subunit of the budding yeast SWI/SNF-family remodeler RSC (Remodeling the Structure of Chromatin), recruits Arp7 and Arp9^15^. In turn, the Arp7/9 heterodimer forms a tight complex with another auxiliary subunit, Rtt102. Biochemical and structural studies have shown that Rtt102 stabilizes a compact conformation of Arp7/9 that favors binding to the HSA domain, while also promoting high-affinity binding of ATP to one of the Arps^23, 24^. Arp7/9 regulates the nucleosome sliding, ejection, and ATPase activities of RSC in an Rtt102-dependent manner^16, 23^. However, the molecular mechanism through which binding of Rtt102-Arp7/9 to the HSA domain of Sth1 regulates its activity is unknown.

Several studies have identified regulatory interactions between the ATPase domain and flanking regions in various catalytic subunits^25–29^. Interestingly, in Sth1, mutations that restore viability of ΔArp7/9 yeast strains localize to two highly conserved regions: protrusion-1 (P1) within the ATPase domain and a ~60-aa sequence in between the HSA and ATPase domains known as the post-HSA (pHSA) domain^15^. It is therefore possible that the pHSA domain of Sth1 interacts with the ATPase domain, and this interaction could in principle be regulated by Rtt102-Arp7/9 binding to the neighboring HSA domain. In this study, we test this hypothesis; we show that the pHSA domain interacts with the ATPase domain, directly contacting P1. We further show that binding of Arp7/9 to the HSA domain weakens this interaction in an Rtt102-dependent manner. In addition, the binding of Rtt102-Arp7/9 to the HSA domain reduces the affinity of the ATPase for DNA, while promoting the formation of stable, monodisperse complexes with both DNA and nucleosomes. A nucleotide-bound structure of Rtt102-Arp7/9 reveals that ATP binds only to Arp7, helping stabilize a closed conformation of Arp7 that potentiates binding of Rtt102-Arp7/9 to the HSA domain. Together, the results suggest a molecular mechanism for how actin and Arps might regulate SWI/SNF-family and INO80-family remodelers.

## Results

### The pHSA and ATPase domains of Sth1 interact with each other

**Fig. 1:**
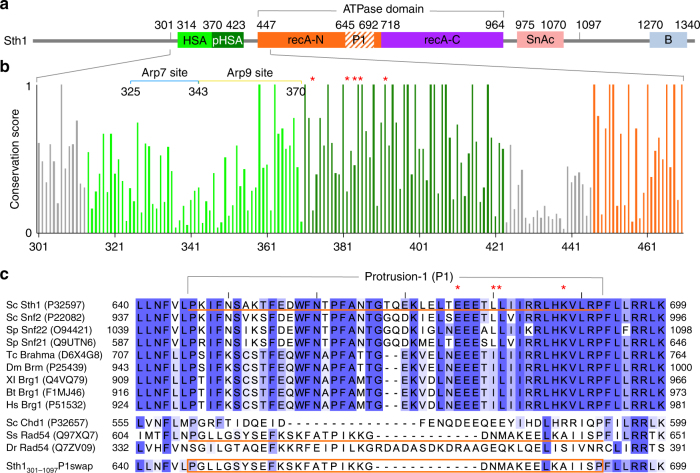
Conservation analysis of the HSA–pHSA and P1 regions of the catalytic subunit of SWI/SNF-family remodelers. **a** Domain architecture of Sth1, the catalytic subunit of the budding yeast RSC remodeler: *HSA* helicase-SANT-associated domain, *pHSA* post-HSA domain, *recA-N and recA-C* N-terminal and C-terminal recA domains of the ATPase, *P1* protrusion-1, *SnAc* Snf2 ATP coupling, *B* bromodomain. **b** Plot of sequence conservation scores calculated from an alignment of nine representative sequences of catalytic subunits from SWI/SNF-family remodelers and plotted for the 301–470 region of Sth1. The binding sites of Arp7 and Arp9 are indicated, and red asterisks designate the sites of Sth1 mutations that restore viability of ΔArp7/9 strains. **c** Sequence alignment of the P1 region for the catalytic subunits of the nine SWI/SNF remodelers used to calculate the conservation scores shown in part (**b**; upper group) and three remodelers that do not bind actin or Arps (lower group). The swapped regions of Sth1 and Rad54 are underlined (orange), and the resulting Sth1_301–1097_P1swap construct is shown at the bottom. Red asterisks designate the sites of Sth1 mutations that restore viability of ΔArp7/9 strains

**Fig. 2:**
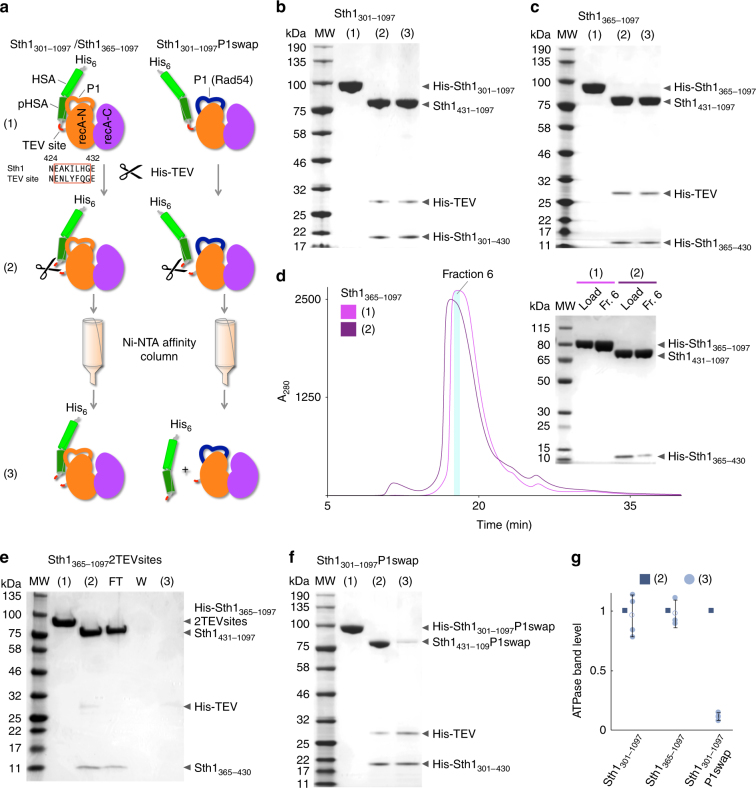
The pHSA domain of Sth1 interacts with P1. **a** Scheme depicting the TEV cleavage and co-purification assay designed here to test for intramolecular interactions between the HSA–pHSA and ATPase regions of Sth1. **b**, **c** SDS-PAGE analysis (4–12% gradient gel) of Sth1_301–1097_ (**b**) and Sth1_365–1097_ (**c**) before cleavage (1), after cleavage (2), and after co-purification (3), as depicted in part (**a**). **d** Analytical size exclusion chromatography of Sth1_365–1097_ before (1) and after (2) TEV cleavage. The inset shows a 4–12% gradient gel of the load (diluted 1:10) and fraction-6 (cyan) for each sample. **e** SDS-PAGE analysis (4–15% gradient gel) of construct Sth1_301–1097_ 2TEV sites (containing two TEV cleavage sites, one after the N-terminal His-tag and another between the HSA and pHSA domains). Samples analyzed: Sth1_365–1097_ 2TEV sites before cleavage (1), Sth1_365–1097_ 2TEVsites after cleavage (2), Ni-NTA flow-through (FT), wash (W), and elution (3). **f** SDS-PAGE analysis (4–12% gradient gel) of construct Sth1_301–1097_P1swap, in which P1 was swapped by that of Rad54 (see Fig. 1c) before cleavage (1), after cleavage (2), and after co-purification (3), as depicted in part (**a**). **g** Dot-plot quantification of the amount of Sth1 ATPase domain associated with the N-terminal His-tagged fragment after TEV cleavage (dark blue squares) and co-purification through the Ni-NTA affinity column (light blue circles). The closed and open shapes represent technical replicate and the mean, respectively. The error bars represent the S.D. from three (Sth1_365–1097_ and Sth1_301–1097_P1swap) or four (Sth1_301–1097_) technical replicates

The HSA domain, defined as the region of the ATPase that binds Rtt102-Arp7/9, and the more highly conserved pHSA domain are unique to actin/Arp-containing remodelers (Fig. 1 and Supplementary Fig. 1). The existence of an intramolecular interaction involving the pHSA domain was first suggested by the observation that construct Sth1_425–1097_, lacking both the HSA and pHSA domains, was insoluble and accumulated in bacterial inclusion bodies, whereas a construct lacking only the HSA domain, Sth1_365–1097_, was soluble. Since Sth1 cannot be expressed without the pHSA domain to test the existence of a hypothetical intramolecular interaction involving the pHSA domain, we designed an internally cleavable construct, whereby the HSA–pHSA region could be severed from the ATPase domain after purification. For this, a canonical TEV protease cleavage site was engineered within the poorly conserved linker between the pHSA and ATPase domains and with minimal changes to the endogenous sequence (Figs 1b and 2a). The resulting construct (TEV-cleavable Sth1_301–1097_) contains a non-cleavable N-terminal His-tag for affinity purification and pull-down assays (Fig. 2a). Purified TEV-cleavable Sth1_301–1097_ was soluble, and remained soluble after cleavage with TEV protease. Analysis by SDS-PAGE confirmed the formation of two fragments, with masses corresponding to the His-HSA–pHSA and ATPase fragments (Fig. 2b and Supplementary Fig. 2). Importantly, the untagged ATPase domain co-eluted with His-HSA–pHSA on a Ni-NTA affinity column (Fig. 2a, b, g). The same results were obtained with TEV-cleavable construct Sth1_365–1097_, lacking the HSA domain; Sth1_365–1097_ remained soluble after TEV cleavage and the resulting untagged ATPase and His-pHSA fragments co-eluted on a Ni-NTA column (Fig. 2c, g). These results suggested that the untagged ATPase domain remains bound to the His-tagged N-terminal fragments through interaction with the pHSA domain. To rule out the possibility that the ATPase domain bound independently to the Ni-NTA affinity column (despite the lack of an affinity tag on this portion of the protein), TEV-cleaved Sth1_365–1097_ was purified through a gel filtration column, which again showed the ATPase and His-pHSA fragments co-migrating as a single peak, as further confirmed by SDS-PAGE analysis (Fig. 2d). In this case, however, we noticed some loss of His-pHSA due to dilution in the column, characteristic of interactions with affinities in the micromolar range^30^. As an added control, we inserted a second TEV site in between the N-terminal His-tag and the pHSA domain of construct Sth1_365–1097_. In this case, TEV protease is expected to cleave at two locations, between the His-tag and pHSA domains and between the pHSA and ATPase domains. As anticipated, after cleavage the untagged pHSA and ATPase fragments flowed through the Ni-NTA column, whereas the non-cleaved His-tagged protein remained bound (Fig. 2e). Taken together, these results support the presence of a direct intramolecular interaction involving the pHSA and ATPase domains of Sth1.

### The pHSA domain interacts directly with protrusion-1

Because mutations that suppress ΔArp7/9 lethality localize to the pHSA and P1 regions of Sth1 (residues highlighted by red asterisks in Fig. 1b, c)^15^, these two regions might be expected to interact with each other. To test this possibility, P1 in the TEV-cleavable construct Sth1_301–1097_ was swapped with P1 from Rad54, a homologous SF2 ATPase that does not bind actin or Arps and therefore has a different P1 (Fig. 1c). In the resulting construct (Sth1_301–1097_P1swap), Sth1 residues P646-P692 were replaced with Rad54 residues P610-P644 (which is 12-aa shorter), such that the swapped region is flanked by conserved sequences that can be unequivocally aligned (Fig. 1c). Importantly, the replaced sequence is mostly exposed in the structure of Rad54^7^, and the few residues that interact with the ATPase domain tend to be conserved in Sth1. The migration profile of the P1 swapped construct on a gel filtration column suggests that the large substitution of 47 amino acids does not affect folding of the ATPase (Supplementary Fig. 3a), further supported by the demonstration of DNA-dependent ATPase activity (Supplementary Fig. 3b) and by the of the swapped construct DNA-binding affinity (Supplementary Fig. 3c), both of which are similar to those of the wild-type protein.

As anticipated, the P1 substitution almost entirely abolished the ability of His-HSA–pHSA to pull-down the untagged ATPase domain on a Ni-NTA affinity column after TEV cleavage (Fig. 2f, g). These results suggest that the pHSA and P1 regions of Sth1 interact directly with each other. Because the pHSA and P1 regions are highly conserved within (albeit not across) the SWI/SNF and INO80 families of remodelers (Fig. 1 and Supplementary Fig. 1), it is possible that this interaction is conserved among actin/Arp-containing remodelers.

### Rtt102-Arp7/9 destabilizes the pHSA–protrusion-1 interaction

**Fig. 3:**
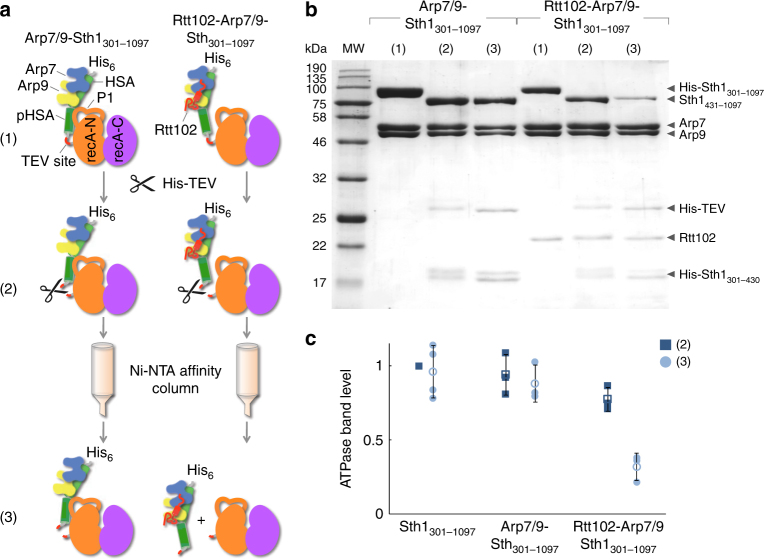
Rtt102-Arp7/9 regulates the interaction between pHSA and P1. **a** Scheme depicting the TEV cleavage and co-purification approach to test the effect of Arp7/9 binding on the interaction between pHSA and P1 ± Rtt102. **b** SDS-PAGE analysis (12% gel) of Sth1_301–1097_ plus Arp7/9 or Rtt102-Arp7/9 before cleavage (1), after cleavage (2), and after co-purification (3), as depicted in part (**a**). **c** Quantification of the amount of the Sth1 ATPase domain associated with N-terminal His-tagged complexes after TEV cleavage (dark blue squares) and co-purification (light blue circles). The closed and open shapes represent each technical replicate and the mean, respectively. The error bars represent the S.D. from three (Arp7/9-Sth1_301–1097_ and Rtt102- Arp7/9-Sth1_301–1097_) or four (Sth1_301–1097_) technical replicates

Since mutations in P1 and the pHSA domain restore the viability of ΔArp7/9 yeast strains^15^, we reasoned that the binding of Arp7/9 to the HSA domain could regulate the pHSA–P1 interaction. To test this possibility, the TEV cleavage and pull-down approach described above were performed with construct Sth1_301–1097_ co-purified with Arp7/9 (Fig. 3a). To our surprise, however, a large fraction of the ATPase domain co-eluted with the Arp7/9-bound His-HSA–pHSA fragment (Fig. 3b, c). Therefore, Arp7/9 alone appears to have little effect on the pHSA–P1 interaction.

Because we previously found that subunit Rtt102 stabilizes the Arp7/9 heterodimer, enhancing its interaction with the HSA domain^24^, we repeated the experiment with Rtt102-Arp7/9 (Fig. 3a). In this case, ~23% of the ATPase was lost after TEV cleavage, likely due to protein precipitation (as mentioned above, in isolation the ATPase domain has low solubility), and an additional 45% was lost after passage through a Ni-NTA affinity column (Fig. 3b, c). These results suggest that the binding of Rtt102-Arp7/9 to the HSA domain weakens the interaction between the pHSA and P1 regions of Sth1.

### Rtt102-Arp7/9 reduces the affinity of Sth1 for DNA

**Fig. 4:**
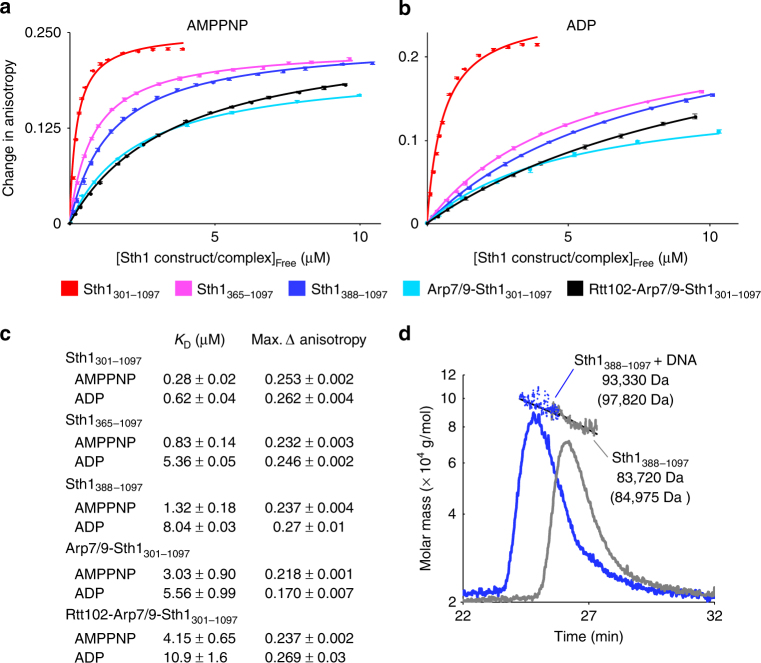
Rtt102-Arp7/9 regulates DNA binding to Sth1. **a**, **b** DNA binding to constructs Sth1_301–1097_, Sth1_365–1097_, Sth1_388–1097_, Arp7/9-Sth1_301–1097_, and Rtt102-Arp7/9-Sth1_301–1097_ measured by fluorescence anisotropy. Each sample was analyzed in the presence of 1 mM AMPPNP (**a**) or 1 mM ADP (**b**). Solid lines represent the fits to a single-site hyperbolic binding isotherm (color-coded per construct or complex as shown). Error bars represent the S.D. from three independent measurements for each experiment. **c** Comparison of the affinities (*K*_D_) and maximum anisotropy changes derived from the fits of the experiments in parts (**a**, **b**). The reported error values represent the S.D. of the fits from three independent experiments. **d** SEC-MALS analysis of the molecular mass of Sth1_388–1097_ with (blue) or without (gray) the 20-bp DNA duplex used in anisotropy experiments (without the fluorescin labels). The theoretical masses are listed in parentheses

The results described above suggested that the binding of Rtt102-Arp7/9 produces a conformational change in Sth1, which in principle could affect DNA binding, since the structure of Rad54 shows P1 making contacts with DNA^7^. To verify this possibility, we measured DNA binding to Sth1 constructs alone and in complex with Rtt102-Arp7/9 using fluorescence anisotropy, as previously described^31^ (Fig. 4). Three Sth1 constructs were tested in these experiments: Sth1_301–1097_, Sth1_365–1097_, lacking the HSA domain, and Sth1_388–1097_, lacking additionally 17-aa of the pHSA domain. The proteins and DNA used in the experiments were purified to homogeneity (Supplementary Fig. 4a, b), and all the Sth1 constructs were soluble, monomeric and formed 1:1 complexes with DNA under the conditions of the assays, as illustrated here for construct Sth1_388–1097_ using multi-angle light scattering (Fig. 4d).

To measure DNA binding in the ATP state, the non-hydrolyzable analog AMPPNP was used to prevent DNA-dependent nucleotide hydrolysis during the experiments. In the AMPPNP-bound state, Sth1_301–1097_ bound DNA with a *K*_D_ of 0.28 μM, whereas Sth1_365–1097_ bound with ~3-fold weaker affinity (*K*_D_ of 0.83 μM; Fig. 4a, c). Since these two constructs differ only in the presence/absence of the HSA domain, their different DNA-binding affinities suggest that the HSA domain contacts the DNA, consistent with the presence of 14 basic residues within this region of Sth1. No functional relevance is assigned here to this HSA–DNA interaction, however, since in the full RSC complex Rtt102-Arp7/9 masks the HSA domain. The 23-aa shorter construct Sth1_388–1097_ displayed an additional ~2-fold drop in affinity for DNA (*K*_D_ of 1.32; Fig. 4a, c). Sth1_388–1097_ lacks a highly conserved segment of the pHSA domain that includes most of the ΔArp7/9 suppressor mutations (Fig. 1), and it is thus noteworthy that this region appears to participate in DNA binding when the Arps are not present. On the other hand, the ternary complex Arp7/9-Sth1_301–1097_ and the quaternary complex Rtt102-Arp7/9-Sth1_301–1097_ displayed dramatic ~11-fold and ~15-fold drops in DNA-binding affinity, respectively, compared to Sth1_301–1097_ alone (*K*_D_’s of 3.0 and 4.2 vs. 0.28 μM; Fig. 4a, c). Because the binding of Rtt102-Arp7/9 to the HSA domain weakens the affinity for DNA further than the deletion of the HSA domain (Fig. 4a, c), nonspecific HSA–DNA interactions are only partially responsible for the drop in DNA-binding affinity observed with the quaternary complex. Therefore, at least two factors appear to explain the weaker DNA-binding affinity of the quaternary complex: (a) masking by Rtt102-Arp7/9 of positively charged amino acids within the HSA region that could nonspecifically contact the DNA and (b) weakening of the pHSA–P1 interaction upon binding of Rtt102-Arp7/9 to the HSA domain.

To test whether Rtt102-Arp7/9 regulates DNA binding throughout the catalytic cycle, similar experiments were performed in the post-hydrolysis, ADP-bound state. All the Sth1 constructs bound DNA less tightly in the ADP than in the AMPPNP state, and a similar trend was observed in that successive N-terminal deletions of Sth1 or the binding of Rtt102-Arp7/9 to the HSA domain weakened the affinity of Sth1 for DNA (Fig. 4b, c).

Combined, these results suggested that Rtt102-Arp7/9 negatively regulates the binding of Sth1 to DNA. To validate this idea, we used two alternative methods. First, we used a native gel-shift assay in which increasing concentrations of Sth1_301–1097_ (or Rtt102-Arp7/9-Sth1_301–1097_) deplete a fixed concentration of free DNA through complex formation both in the AMPPNP-bound and ADP-bound states. This assay revealed a similar trend to that observed by fluorescence anisotropy; higher concentrations of Rtt102-Arp7/9-Sth1_301–1097_ than Sth1_301–1097_ were required to deplete the DNA band, indicative of a lower DNA-binding affinity of the quaternary complex (Supplementary Fig. 5a–c). This experiment also suggested that the quaternary complex Rtt102-Arp7/9-Sth1_301–1097_ forms a more stable (or homogenous) complex with DNA than Sth1_301–1097_ alone, as indicated by a single, slowly migrating band in the native gel (Supplementary Fig. 5a, b). In the second approach, we used a heparin column to mimic DNA binding. Consistent with the results described above, Sth1_301–1097_ displayed longer retention times on the heparin column than the quaternary complex Rtt102-Arp7/9-Sth1_301–1097_ (Supplementary Fig. 5d).

### Rtt102-Arp7/9-Sth1 stabilizes the Sth1–nucleosome complex

**Fig. 5:**
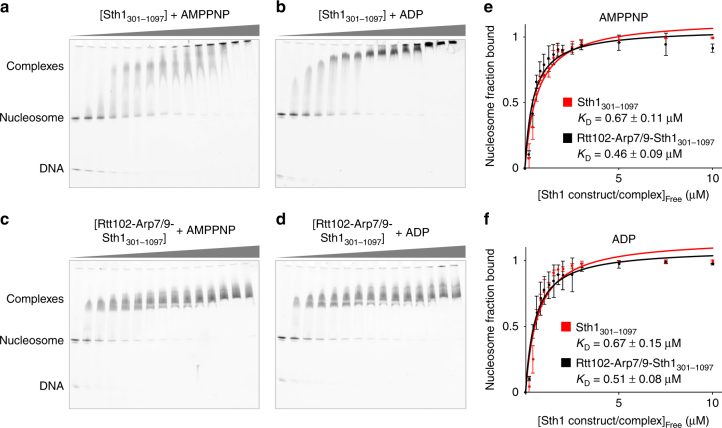
Effect of Rtt102-Arp7/9 on the interaction of Sth1 with nucleosomes. **a**–**d** Native PAGE (4–20% gradient) analysis of nucleosome binding to increasing concentrations of Sth1_301–1097_. The experiments were performed in the presence of 1 mM AMPPNP (**a**, **c**) or 1 mM ADP (**b**, **d**). The Sth1 concentration (μM) increases from left to right as follows: 0, 0.2, 0.4, 0.6, 0.8, 1.0, 1.25, 1.5, 1.75, 2.0, 2.5, 3.0, 5.0, 7.5, and 10. The lower band corresponds to free 601 DNA, which was not incorporated into nucleosomes during the assembly reaction. **e**, **f** Fits of the gel-shift data to single-site hyperbolic isotherms, shown separately for the AMPPNP and ADP states. Error bars represent the S.D. of the mean from three technical replicates of each experiment. The errors reported correspond to the S.E. of the fits

In light of the negative effect of Rtt102-Arp7/9 on the affinity of Sth1 for DNA, we asked whether nucleosome binding was similarly affected. Nucleosome binding was measured for Sth1_301–1097_ and Rtt102-Arp7/9-Sth1_301–1097_ in the AMPPNP-bound and ADP-bound states using the native gel-shift assay performed above, in which macromolecules migrate according to their mass/charge ratio. Overall, the complex of Rtt102-Arp7/9-Sth1_301–1097_ with nucleosomes displayed a narrower distribution in native gels compared to that of Sth1_301–1097_ (Fig. 5a–d), which is indicative of a more ordered or compact complex, as also observed with DNA (Supplementary Fig. 5a, b). On the other hand, the nucleotide state did not appear to affect the nucleosome-binding affinities of Sth1_301–1097_ or Rtt102-Arp7/9-Sth1_301–1097_ (Fig. 5e, f). Importantly, Sth1_301–1097_ and Rtt102-Arp7/9-Sth1_301–1097_ bound nucleosomes with similar affinities (unlike their very different affinities for DNA), with a slight albeit reproducible advantage for the quaternary complex both in the AMPPNP (apparent *K*_D_ of 0.46 vs. 0.67 μM) and ADP (apparent *K*_D_ of 0.51 vs. 0.67 μM) states. Therefore, we conclude that, while the isolated Sth1 catalytic subunit and the Rtt102-Arp7/9-Sth1 quaternary complex have similar affinities for nucleosomes, Rtt102-Arp7/9 appears to confer specificity upon Sth1 by promoting the formation of monodisperse complexes with nucleosomes. These results are consistent with previous findings that the quaternary complex has lower DNA-dependent ATPase activity but increased translocation efficiency on nucleosomes^16^.

### The structure of Rtt102-Arp7/9 reveals ATP bound to Arp7

The quaternary complex of Rtt102-Arp7/9-Sth1_301–1097_ studied here contains three potential ATP-binding sites, on Sth1, Arp7, and Arp9, such that nucleotide-dependent effects could in principle be due to ATP binding and/or hydrolysis at any of these sites or their combinations. While ATP binding and hydrolysis by actin has been extensively studied^32^, little is known about the role of ATP in nuclear Arps^33^. In a previous study, we observed using isothermal titration calorimetry that ATP binds to a single, high-affinity site within Rtt102-Arp7/9^24^, whose location we could not precisely identify. Now that we have found that Rtt102-Arp7/9 affects the interaction of Sth1 with nucleosomes, it is imperative to better understand this regulatory complex, including the location and role of ATP in Rtt102-Arp7/9.

**Fig. 6:**
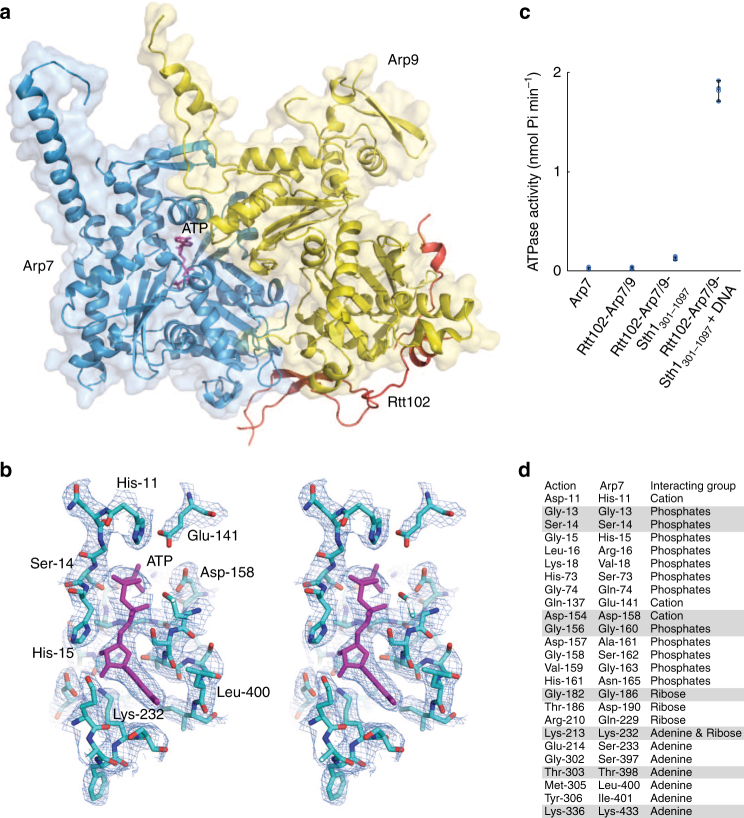
Arp7 binds but does not hydrolyze ATP within the Rtt102-Arp7/9 complex. **a** Crystal structure of the ATP-bound ternary complex of Rtt102-Arp7/9. **b** Wall-eyed stereo view of the ATP-binding pocket of Arp7. Also shown is a composite 2Fo–Fc omit map (blue mesh) contoured at 1.1*σ*. **c** ATPase activities of Arp7, Rtt102-Arp7/9, and Rtt102-Arp7/9-Sth1_301–1097_ (±DNA). The closed and open shapes represent technical replicate and the mean, respectively. The error bars represent the S.D. from three technical replicates. **d** Comparison of the residues in the ATP-binding clefts of actin and Arp7

Reported structures of Arp7/9 (PDB accession code: 3WEE)^34^ and Rtt102-Arp7/9-HSA (PDB accession code: 4I6M)^23^ do not contain ATP bound, presumably because these complexes were crystallized in the presence of high concentrations of phosphate and sulfate ions, which occupy the nucleotide-binding pockets of Arp7 and Arp9. Here, we pre-incubated Rtt102-Arp7/9 with ATP prior to crystallization, and obtained crystals using polyethylene glycol 3350 as the main precipitant. The crystals, which diffracted to a relatively low resolution (3.25 Å), contain two copies of the Rtt102-Arp7/9 ternary complex in the P1 unit cell (RMSD = 0.51 Å for 833 equivalent Cα atoms; Fig. 6a and Table 1). The overall structure, and specifically the disposition of the Arps relative to one another, is marginally closer to that of the quaternary complex Rtt102-Arp7/9-HSA (RMSD of 0.64 Å for 774 equivalent Cα atoms) than that of the Arp7/9 heterodimer (RMSD of 0.67 Å for 758 equivalent Cα atoms). The conformation of Rtt102 is also very similar to that observed in the complex of Rtt102-Arp7/9-HSA, and, although the current structure reveals 13 more amino acids for this subunit, a large portion of Rtt102 (90 amino acids out of 157) remains unresolved in the electron density map. The observable portion of Rtt102 interacts mostly with Arp9, and seems to act as a “clip” holding the Arps together, which may explain why Rtt102 enhances the affinity of Arp7/9 for both Sth1 and nucleotide^24^. More importantly, unlike in previous structures, composite omit maps of the current structure clearly showed the presence of ATP in the nucleotide-binding cleft of Arp7 but not Arp9 in both complexes of the unit cell (Fig. 6b). The striking similarity of the structures suggests that while lacking ATP the two previous structures^23, 34^ displayed a conformation corresponding to that of the nucleotide-bound state, likely due to the presence of phosphate ions in the catalytic cleft substituting for ATP.

**Table 1:** Data collection and refinement statistics

	Rtt102-Arp7/9 (PDB code: 5TGC)
Data collection	
Space group	P1
Cell dimensions	
*a*, *b*, *c* (Å)	79.45, 88.03, 105.49
α, β, γ (°)	109.02, 104.65, 96.20
Resolution (Å)	40.0–3.25 (3.42–3.25)^a^
*R*_merge_ (%)	9.7 (41.8)
*I/*σ(*I)*	11.2 (2.4)
Completeness (%)	96.6 (92.0)
Redundancy	3.4 (2.7)
Refinement	
Resolution (Å)	39.37–3.25 (3.36–3.25)
No. of reflections	38,745
*R*_work_ (%)*/R*_free_ (%)	27.4/31.9
No. of atoms	
Protein	14,131
Ligand/ion (ATP/SO^4−^)	77
*B* factors (Å^2^)	
Protein	70.7
Ligand/ion	72.1
R.m.s deviations	
Bond lengths (Å)	0.004
Bond angles (°)	1.020

The structure was determined using data collected from a single crystal

^a^Values in parentheses are for highest-resolution shell

Monomeric actin is an extremely slow ATPase, and nucleotide hydrolysis is only activated upon polymerization^35^. Not surprisingly, we found here that Arp7 lacks ATPase activity, both alone and within the ternary complex Rtt102-Arp7/9, whereas the quaternary complex Rtt102-Arp7/9-Sth1_301–1097_ displays strong DNA-dependent ATPase activity catalyzed by subunit Sth1 (Fig. 6c). This is consistent with previous findings that mutagenesis of the nucleotide-binding clefts of Arp7 and Apr9 had no functional effect^36^. The structure determined here offers clues that might explain the lack of ATPase activity of Arp7. Out of 26 residues that either directly interact with or fall near the nucleotide, and may thus coordinate water molecules that interact with the nucleotide, only 9 are conserved between actin and Arp7 (Fig. 6d). In particular, the all-important actin residue Lys-18, which anchors the nucleotide in place by interacting with both the α-phosphate and β-phosphate, becomes Val-18 in Arp7, which cannot support these interactions. In actin, the β-phosphate and γ-phosphate of the nucleotide are additionally coordinated by a divalent cation (Mg^2+^ under physiological conditions, or Ca^+2^ in most of the available structures). In contrast, there is no cation associated with the nucleotide in the structure of Arp7. Indeed, Arp7 appears to lack a cation-binding site, as two of the residues that coordinate water molecules that hold the cation in place in actin, Asp-11 and Gln-137, have changed in Arp7 to His-11 and Glu-141, respectively. Specifically, Gln-137 and the cation coordinate a water molecule thought to serve as the nucleophile in the ATP hydrolysis reaction of actin. Strikingly, mutating this residue to glutamic acid in yeast actin (the amino acid found in Arp7) results in lack of viability^32^. The same study found that residue His-161 in the actin cleft is also absolutely required for yeast viability. Actin His-161 becomes Asn-165 in Arp7. Therefore, the lack of ATPase activity in Arp7 is supported by the structure and previous genetic studies on actin and the Arps^36^.

## Discussion

We have shown here that the pHSA domain of Sth1 interacts with the P1 region of the ATPase domain, and that regulation of this interaction might be an important mechanism to fine-tune the activity of actin/Arp-containing remodelers. This finding adds to a growing list of evidence showing that domains adjacent to the ATPase domain regulate the activity of the catalytic subunits of several remodelers. Thus, N-terminal chromodomains in Chd1 interact with and lock the ATPase domain in an inactive conformation^26^. The AutoN and NegC regions of ISWI, N and C terminal to the ATPase domain, negatively regulate ATP hydrolysis and the link between ATP hydrolysis and productive DNA translocation, respectively^25^. Similarly, the N-terminal region of CSB negatively regulates its ATPase activity^27^. In SWI/SNF remodelers, the SnAc (Snf2 ATP coupling) domain positively regulates the catalytic activity of the ATPase domain and may also interact with histones^29, 37^.

During the preparation of this manuscript, a structure of the Sth1 homolog Snf2 from *Myceliophthora thermophila* was reported^38^. The structure comprises Snf2 residues Ala-458 to Gly-1128, corresponding to Sth1 residues Ser-383 to Gly-1049 (i.e., it does not include the HSA domain). Although several elements of the structure were not visualized, including parts of P1 and the linker between the pHSA and ATPase domains, the structure offers important clues; it reveals most of the pHSA domain (equivalent to Sth1 residues Ser-383 to Val-419) and a portion of the C-terminal SnAc domain (equivalent to Sth1 residues Asp-971 to Gly-1049). Both the SnAc and pHSA domains interact extensively with the ATPase domain and, consistent with our findings here, the pHSA domain lies adjacent to P1. The concurrence of our findings and the high conservation of the interacting sequences in the individual SWI/SNF and INO80 families of remodelers (Fig. 1 and Supplementary Fig. 1) allow us to postulate that the pHSA–P1 interaction is conserved throughout actin/Arp-containing remodelers.

A recent study found that the binding of Rtt102-Arp7/9 increases the activity of Sth1 by allowing for more efficient translocation per ATP hydrolysis cycle^16^. The same study found that a ΔArp7/9 yeast viability-restoring mutation in the P1 region had the same effect in the absence of Rtt102-Arp7/9. This raises the question as to how the suppressor mutations restore viability of ΔArp7/9 yeast strains? Our initial thought was that these mutations had the same effect as the binding of Rtt102-Arp7/9 to the HSA domain of the catalytic subunit, namely weakening the interaction between the pHSA and P1 regions. However, this does not appear to be the way these mutations work. Indeed, several of the mutated amino acids were visualized in the recent structure of Snf2^38^, and appear scattered throughout the pHSA and P1 regions, not forming a single cluster. What is more, the suppressor mutations are not found within the hydrophobic interface between the pHSA and P1 regions, and instead point away from this interface, and thus these mutations are not expected to affect the pHSA–P1 interaction. Therefore, a mechanistic understanding of the effect of the suppressor mutations is still lacking.

Given the intrinsic structural flexibility of the recA domains relative to one another^7, 8, 26, 38, 39^, it is possible that by breaking the interaction between the pHSA and P1 regions the role of Rtt102-Arp7/9 is to free the recA domains to adopt a conformation more suitable for DNA translocation on nucleosomes, as recently suggested^16^. Likely, the linker between the HSA and pHSA helices, which contains three of the yeast viability-restoring mutations (Fig. 1), serves as a hinge for Rtt102-Arp7/9-dependent conformational changes in Sth1. In this regard, it is important to note that a recent cryo-EM structure of the Sth1-related catalytic subunit Snf2 from *Saccharomyces cerevisiae* bound to the nucleosome shows an unexpected new role of the P1 region (called SuppH in the cited work); it participates in inter-recA domain stabilizing interactions^39^. Thus, two helices of the P1 region within the N-terminal recA domain fold upon two helices of the so-called “Brace” region within the C-terminal recA domain in a crossed-arms manner to stabilize the recA domains around the nucleosome. As a result, the inter-recA conformation observed in this structure is very different from that observed in the absence of nucleosome^38^. Yet, the pHSA helix lies adjacent to the P1 region in both structures, suggesting that they move as a single entity. If, as our work suggests, the binding of Rtt102-Arp7/9 to the neighboring HSA domain weakens the pHSA–P1 interaction, this could impact the orientation of the recA domains with respect to one another and thus their interaction with the nucleosome, which could possibly explain the formation of the monodisperse Rtt102-Arp7/9-Sth1/nucleosome complex observed here.

The structure of ATP-bound Rtt102-Arp7/9 described here and previous biochemical data^24^ confirm the presence of a nucleotide-binding site in Arp7. However, we also found that Rtt102-Arp7/9 lacks ATPase activity (Fig. 6c), suggesting that ATP binding to Arp7 plays a regulatory role. And yet, ATP is likely bound under physiological conditions, since the concentration of ATP in the nucleus is ~3.5 mM^40^ and Rtt102-Arp7/9 binds ATP with a *K*_D_ of 200 nM, and even higher affinity when bound to the HSA domain^24^. What is then the role of ATP binding to Arp7? Comparison with a recent structure of actin/Arp4 bound to the HSA domain of SWR1^41^ offers a potential explanation. In this structure, Arp4 occupies the position of Arp7, at the N-terminal end of the HSA domain, whereas actin takes the position of Arp9. Remarkably, Arp4 displays a closed-cleft conformation and contains ATP bound, whereas actin shows an open cleft conformation and lacks nucleotide (just like Arp9 in the structure determined here). There are >100 actin entries in the Protein Data Bank, and to our knowledge the actin/Arp4-SWR1 structure is the first in which actin has no nucleotide bound. In contrast, Arp7, which in isolation has low affinity for ATP (*K*_D_ = 12 μM), binds nucleotide tightly (*K*_D_ = 94 nM) as part of the quaternary complex Rtt102-Arp7/9-HSA^24^. Thus, there appears to be a conserved structural requirement for a closed, nucleotide-bound cleft for the subunit associated with the N-terminal half of the HSA domain and an open, nucleotide-free cleft for the subunit bound closer to the ATPase domain. In other words, we propose that the role of nucleotide binding to actin/Arp-containing remodelers is not regulatory, but structural. If this prediction is correct, it could be expected that the fission yeast remodelers RSC and SWI/SNF, which contain Arp9 and the Arp4 homolog Arp42 (instead of Arp7)^42^, will have nucleotide-bound Arp42 and nucleotide-free Arp9 at the N-terminal and C-terminal ends of the HSA domain, respectively.

In summary, the biochemical and structural data presented here suggest that Rtt102 and ATP stabilize a conformation of Arp7/9 that potentiates binding to the HSA domain, which in turn releases intramolecular interactions between the pHSA domain and the P1 region of the ATPase domain. These interactions might control the affinity and specificity of the ATPase for DNA and nucleosomes, ultimately regulating the translocation efficiency of the remodeler. Because both the pHSA domain and P1 are highly conserved separately within the SWI/SNF and INO80 remodeler families, this could represent a general mechanism for how actin and Arps regulate the activities of their host remodelers.

## Methods

### Proteins

Sth1 constructs and complexes were cloned and expressed as described^24^. For Sth1_301-1097_P1Swap, the Sth1 fragments 301–645 and 693-1097 were primer-extended to add *S. solfataricus* Rad54 residues 610–644. A silent EcoRI site was introduced during extension to ligate the two Sth1 fragments. For protein purification, cells were resuspended in lysis buffer (20 mM HEPES pH 7.5, 300 mM NaCl, 5% glycerol, 25 mM imidazole, and 4 mM benzamidine), lysed using a Microfluidizer apparatus (Microfluidics), and clarified by centrifugation. Lysates were purified on a Ni-NTA affinity column (Qiagen) and washed extensively with lysis buffer. Proteins were eluted with 250 mM imidazole and bound to a HiTrap Heparin HP column (GE Healthcare) equilibrated in sample buffer (20 mM HEPES pH 7.5, 200 mM NaCl, 5% glycerol, 2 mM dithiothreitol (DTT), and 4 mM benzamidine). Proteins were eluted using a 200–800 mM NaCl gradient, and further purified on a SD-200 gel filtration column (GE Healthcare) equilibrated with sample buffer. To reconstitute the Rtt102-Arp7/9 ternary complex, Rtt102 and Arp7/9^24^ were mixed at a 2:1 molar ratio and purified through a SD-200 gel filtration column equilibrated in sample buffer (without benzamidine).

### TEV cleavage and co-purification assay

A TEV site was engineered between Sth1 residues 424 and 432 by site-directed mutagenesis (Quikchange, Agilent). Purified Sth1 constructs at 6 μM (±Arp7/9 and Rtt102) were incubated with 20 μg of TEV protease overnight in co-purification buffer (20 mM HEPES pH 7.5, 500 mM NaCl, 5% glycerol, 25 mM imidazole, 1 mM MgCl_2_, and 0.1 mM AMPPNP). A 1-mL volume of the reaction was then incubated for 4 h with 250 μL of Ni-NTA resin (Qiagen) equilibrated in co-purification buffer. Unbound protein was flowed through a PolyPrep column (Bio-Rad), and the resin was washed with 5 mL of co-purification buffer, followed by elution with 250 mM imidazole. The samples were then analyzed by SDS-PAGE, and the Coomassie-stained bands were quantified densitometrically using the program ImageJ^43^. For experiments with Sth1 alone, the ATPase level after cleavage was set to 1. To account for loading differences (before and after co-purification), the loss of ATPase after co-purification was calculated by normalizing the intensities of the His-tagged N-terminal fragments and adjusting the intensities of the ATPase bands accordingly. For experiments with Arp7/9, the ATPase levels were normalized using the intensities of Arp7 and Arp9 as an internal reference. The normalized ATPase band intensities for the TEV-digested samples were divided by 0.91, a factor accounting for the loss of intensity of the ATPase band upon TEV cleavage.

### Analytical size exclusion chromatography

Sth1_365–1097_ at 200 μM was digested in column buffer (20 mM HEPES pH 7.5, 500 mM NaCl, 5% glycerol, 2 mM DTT) with 100 μg TEV protease. A volume of 500 μL of the digested sample was injected onto a Bio-Sil SEC 250 HPLC column (Bio-Rad) at a flow rate of 0.5 mL/min, and 0.25 mL fractions were collected starting at 15 min post injection. As a control, 500 μL of undigested Sth1, supplemented with an equivalent amount of TEV purification buffer, was also analyzed.

### Oligonucleotide preparation

Oligos (5′-TCCATGTCCATGGATACGTGG-3′ and 5′-TCCACGTATCCATGGACATGGA-3′) were injected onto a Symmetry300 C18 column (Waters) equilibrated with 0.1 M TEAA pH 7.0 and purified on a 0–20% acetonitrile gradient. Following cycles of lyophilization and resuspension in 50% ethanol to fully remove the TEAA and acetonitrile, the oligos were resuspended in hybridization buffer (20 mM HEPES pH 7.5, 2.5 mM MgCl_2_ and 50 mM NaCl). Equimolar (100 μM) amounts of each strand were mixed at 95 °C and annealed by slow cooling to room temperature. For anisotropy experiments, the oligos contained a fluorescein label at the 5′-end.

### Fluorescence anisotropy

Prior to titrations, a 1.5 mL volume of the double-stranded oligonucleotide (50 nM) was equilibrated in hybridization buffer (supplemented with 5% glycerol, 1 mM DTT, 1 mM AMPPNP or ADP, and 0.1 mg/mL bovine serum albumin (BSA)) for 30 min at 20 °C. The anisotropy of free DNA was recorded with a Cary Eclipse fluorescence spectrophotometer (Varian) by excitation at 485 nm and recording the average anisotropy between 515 and 520 nm. Measurements were performed in triplicate. Sth1 (±Rtt102-Arp7/9) at ~20–40 μM was titrated, and recordings were taken after 4 min of stirring. The changes in anisotropy were obtained by subtracting the anisotropy of free DNA from that of DNA–protein complexes. Data were fitted to a single-site, hyperbolic binding isotherm with the program Igor (WaveMetrics) to obtain dissociation constants.

### Size exclusion chromatography–multi-angle light scattering

Sth1_388–1097_ at 100 μM was incubated with a 1.5-fold molar excess of the double-stranded oligo (described above) for 2 h at 20 °C. The complex was loaded onto a TSKgel SuperSW2000 column (Tosoh Bioscience) connected inline with a DAWN HELEOS MALS detector and an Optilab rEX refractive index detector for mass analysis. Molecular masses were calculated with the program Astra (Wyatt Technology).

### Native gel-shift assays

Histones were assembled and purified using the salt gradient dialysis method as previously described^44^. Briefly, *Xenopus laevis* core histones were expressed in BL21 Rosetta cells and purified from inclusion bodies. Histone octamers were reconstituted and purified by gel filtration chromatography. To determine the optimal ratio for nucleosome assembly, histone octamers were mixed with varying concentrations of 601 nucleosomal DNA for test assemblies. Nucleosomes were assembled according to the optimal DNA:histone ratio and purified by gel filtration chromatography.

Native gel-shift assays were performed in 20 mM HEPES pH 7.5, 50 mM NaCl, 5% glycerol, 1 mM DTT, 0.1 mg/mL BSA, 1 mM MgCl^2^, and 1 mM ADP (or AMPPNP). The concentration of nucleosomes or free DNA was held constant at 50 μM, and Sth1 constructs were added at a range of concentrations to a maximum of 10 μM. The reactions were incubated for 30 min at room temperature, and 10 μL aliquots were loaded onto a 4–20% TBE gel (Bio-Rad) in 0.5 × TBE. The gels were run at 4 °C and 150 V for 3 h (nucleosome) or 45 min (free DNA), and then stained with SYBR Gold (GE Healthcare) and imaged on a Bio-Rad Gel Doc Imager. The bands corresponding to the nucleosome core particle or the 21-bp DNA duplex were quantified densitometrically using the program ImageJ. For each lane, the nucleosome band was quantified and normalized to the no-Sth1 control, and these values were subtracted from 1 to obtain the fraction of nucleosomes bound.

### Crystal structure of Rtt102-Arp7/9

Rtt102-Arp7/9 at 10 mg/mL was incubated with 1 mM ATP for 30 min, and then crystallized at 16 °C using the sitting drop method. Needle-like crystals were obtained in drops consisting of a 1:1 (v/v) mixture of protein and well solution (20% (v/v) PEG 3350 and 200 mM ammonium sulfate). These initial crystals were used to streak-seed hanging drops consisting of a 1:1.2 (v/v) mixture of protein and well solution (20% (v/v) PEG 3350, 170 mM ammonium sulfate, and 17 mM EDTA). The resulting larger crystals were flash-frozen in liquid nitrogen, using as cryo-protectant 25% glycerol added to the crystallization solution.

X-ray data sets were collected at 100 K on a Bruker × 8 Prospector X-ray diffraction system, fitted with an IμS microfocus-sealed-tube X-ray source with a wavelength of 1.5418 Å, Apex II CCD detector, 4-circle Kappa goniometer, and an Oxford Cryostream 700 liquid nitrogen-cooling system. The diffraction data sets were indexed and scaled with the Bruker program SAINT (version v8.34a). A molecular replacement solution was obtained with the program Phenix^45^, using as a search model PDB entry 4I6M (the HSA domain was not included in the search). Model building and refinement were performed with the programs Coot^46^ and Phenix. The use of the program Rosetta^47^ in combination with Phenix significantly accelerated the convergence of the refinement during the initial steps. The Ramachandran plot of the final model revealed 97.0 and 0.12 percent of resides in favored and outlier regions, respectively. Composite omit maps (Fig. 6b) were calculated with the program Phenix, using the Refine option to remove model bias.

### ATPase assay

Proteins at 5 nM were incubated with 1 mM ATP for 20 min at 22 °C in 20 mM HEPES pH 7.0, 50 mM potassium acetate, 2.5 mM MgCl_2_, 0.1 mg/mL BSA, 200 μM MESG, and 1 unit of purine nucleoside phosphorylase. The reactions were stopped by addition of 250 mM EDTA, and the phosphate release was determined by measuring the absorbance at 360 nm^48^. For Sth1 constructs and complexes, the experiment was performed ±30 μM (nucleotide concentration) of the plasmid pRSF-Duet-1 (Novagen).

### Data availability

The atomic coordinates and structure factors for the ATP-bound Rtt102-Arp7/9 complex described in this study have been deposited in the Protein Data Bank (accession code: 5TGC). The data that support the findings of this study are available from the corresponding author upon request.

## Electronic supplementary material

Supplementary Information
