# Supplementary material for: Actin-related proteins regulate the RSC chromatin remodeler by weakening intramolecular interactions of the Sth1 ATPase
Source: Commun Biol. 2018 Jan 22;1:1. doi: 10.1038/s42003-017-0002-6 (PMC5969521; doi:10.1038/s42003-017-0002-6)
Supplement: Supplementary file 1 — Supplementary Information [file 42003_2017_2_MOESM1_ESM.pdf]

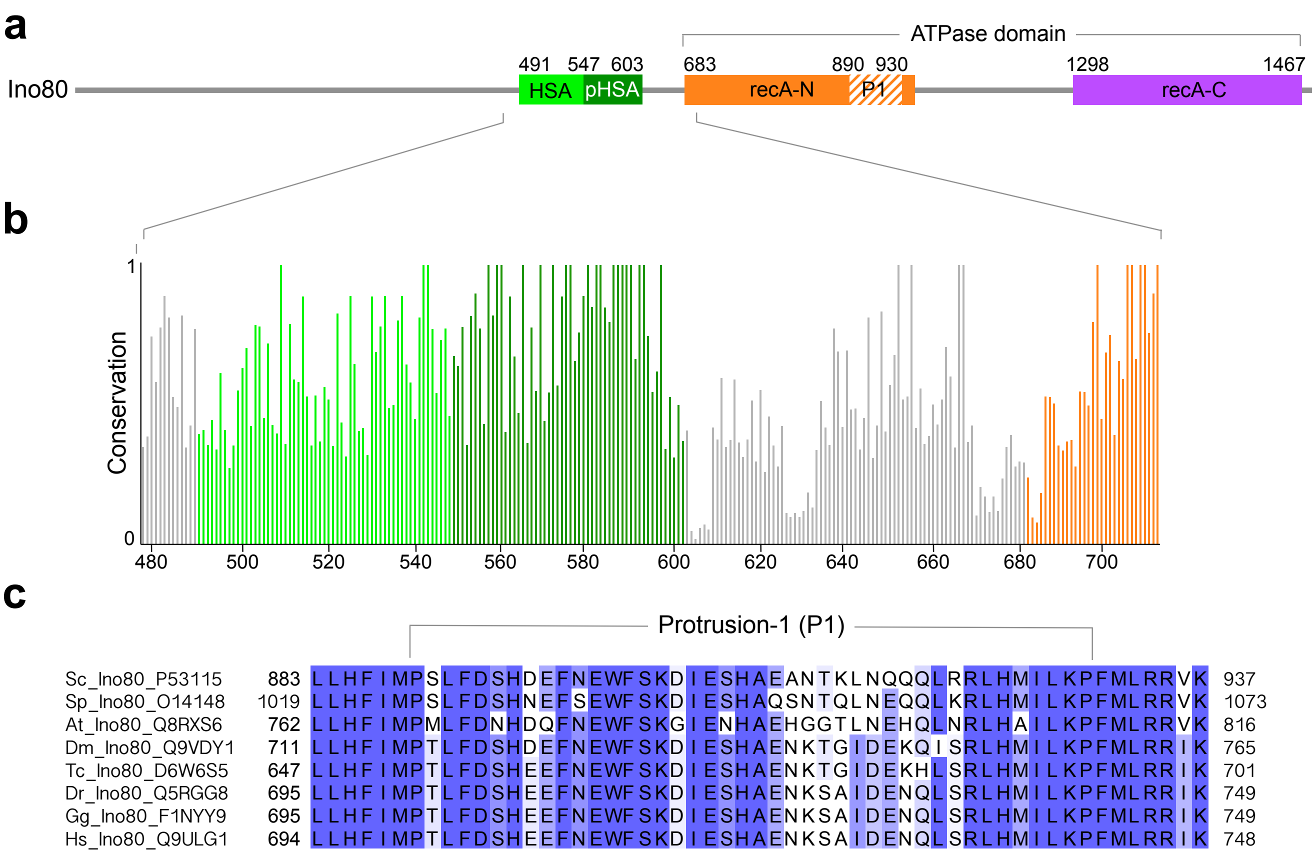

Supplementary Figure 1: Conservation analysis of the HSA-pHSA and P1 regions of the catalytic subunit of INO80-family remodelers. (**a**) Domain architecture of Ino80, the catalytic subunit of the budding yeast INO80 remodeler: HSA, helicase-SANT-associated domain; pHSA, post-HSA domain; recA-N and recA-C, N- and C-terminal recA domains of the ATPase; P1, protrusion-1. (**b**) Plot of sequence conservation scores calculated from an alignment of eight representative sequences of the catalytic subunits of INO80-family remodelers and plotted for the 478-714 region of *Saccharomyces cerevisiae* Ino80. (**c**) Sequence alignment of the P1 region of the catalytic subunits of the eight INO80 remodelers used to calculate the conservation scores in part **b**.

Fig. 2b

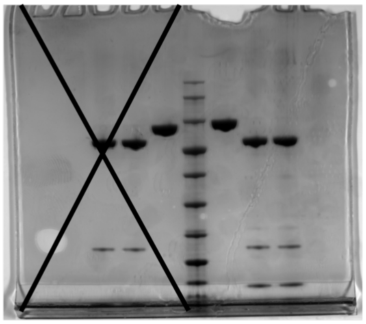

Fig. 2c

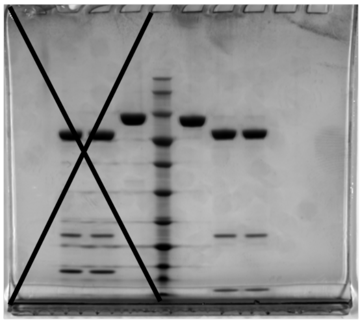

Fig. 2d

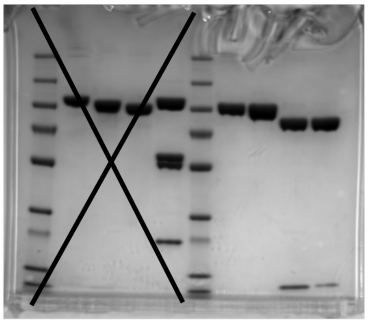

Fig. 2e

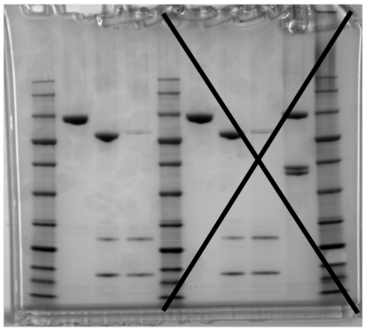

Fig. 3b

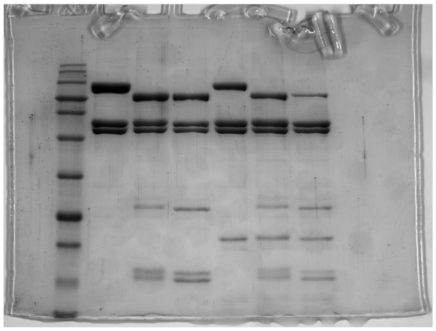

Fig. 5a

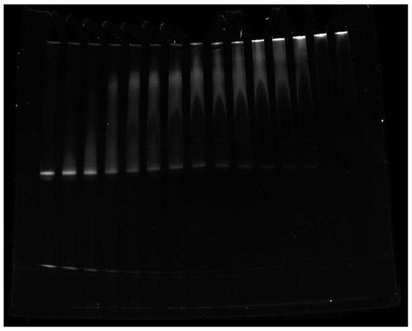

Fig. 5b

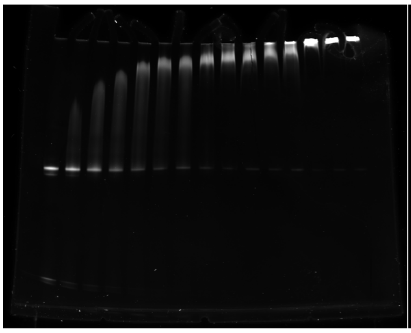

Fig. 5c

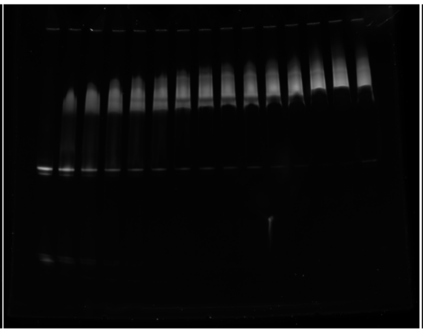

Fig. 5d

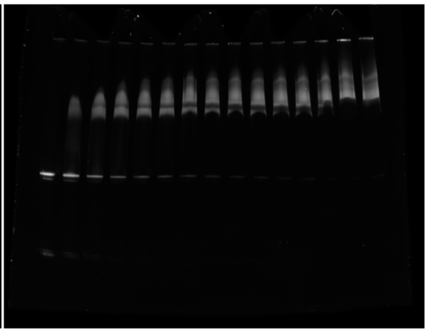

Supplementary Fig. 4a

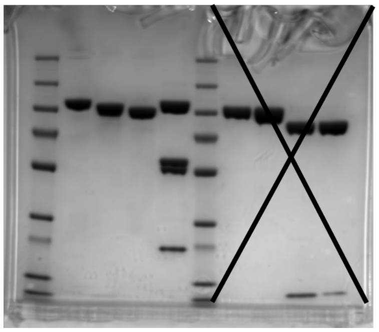

Supplementary Fig. 5a

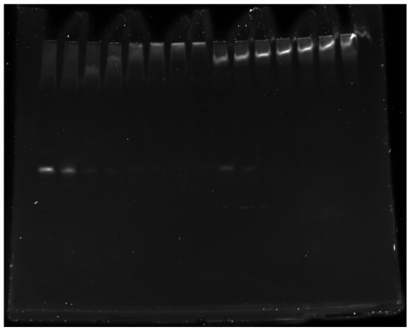

Supplementary Fig. 5b

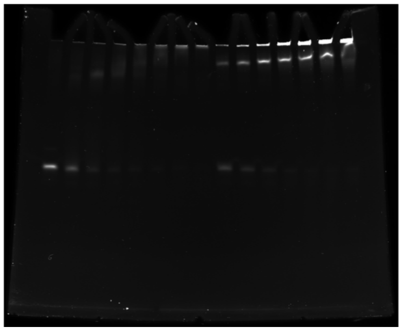

Supplementary Figure 2: Full images of unedited gels. Images of the unedited and uncropped gels used in the main-text the figures indicated above each gel. Sections of the gels not included in the main-text figures are crossed out.

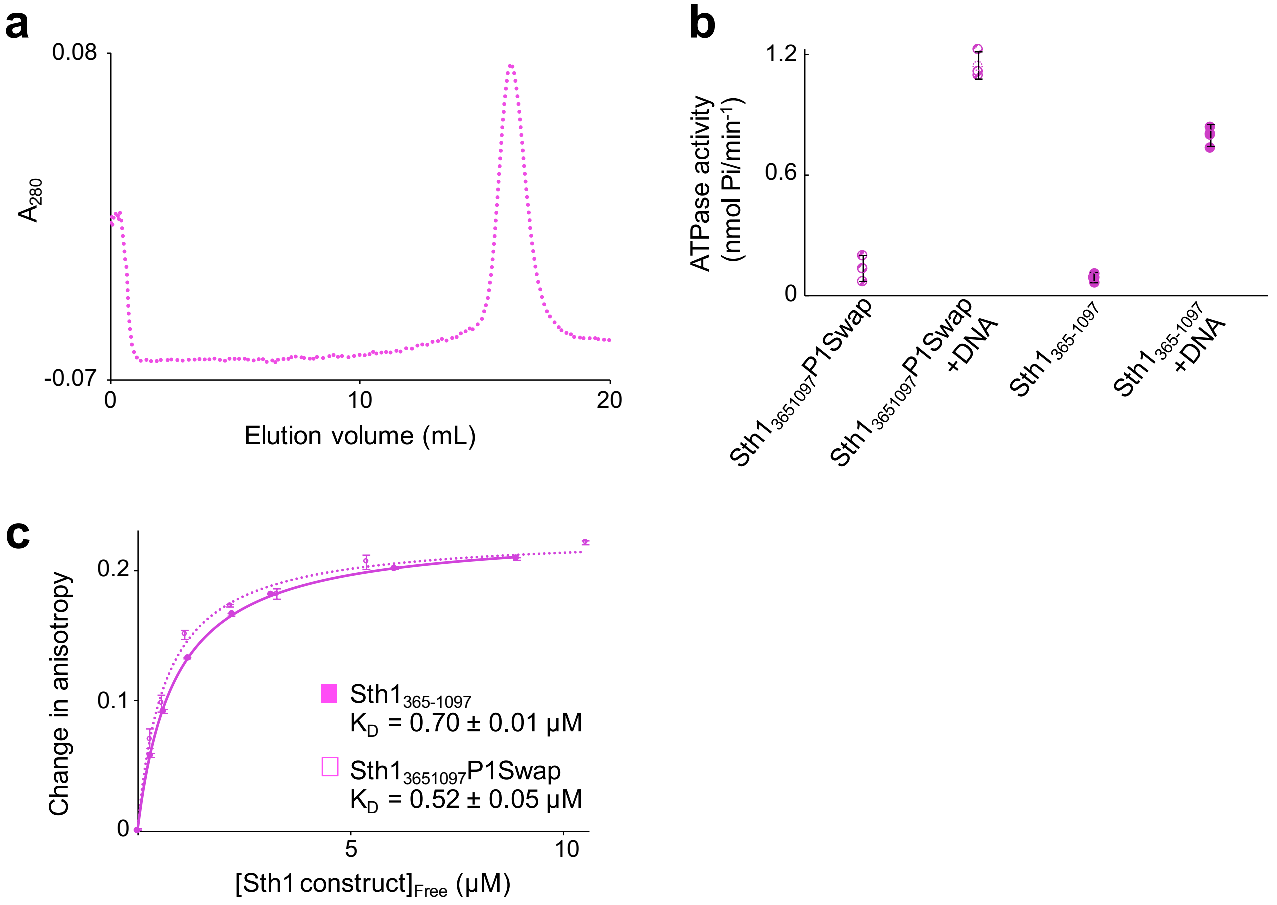

Supplementary Figure 3: Swapping the P1 region of Sth1 for that of Rad54 does not impair the folding of Sth1. (**a**) Elution profile of Sth1$_{365\text{-}1097}$P1Swap on a Superose-6 size-exclusion column. Approximately 1 mg of protein was loaded onto the column in 20 mM HEPES pH 7.5, 500 mM NaCl, 5% glycerol, 2 mM DTT. (**b**) ATPase activities of Sth1$_{365\text{-}1097}$P1Swap and Sth1$_{365\text{-}1097}$ (± DNA). The filled and unfilled circles correspond to the technical replicates and mean value, respectively. The error bars represent the standard deviation from three technical replicates. (**c**) DNA binding to Sth1$_{365\text{-}1097}$P1Swap and Sth1$_{365\text{-}1097}$ measured by fluorescence anisotropy. The experiments were performed in the presence of 1 mM AMPPPNP. Error bars represent the standard deviation from three measurements for each experiment, and the reported error values represent the standard error of the fits.

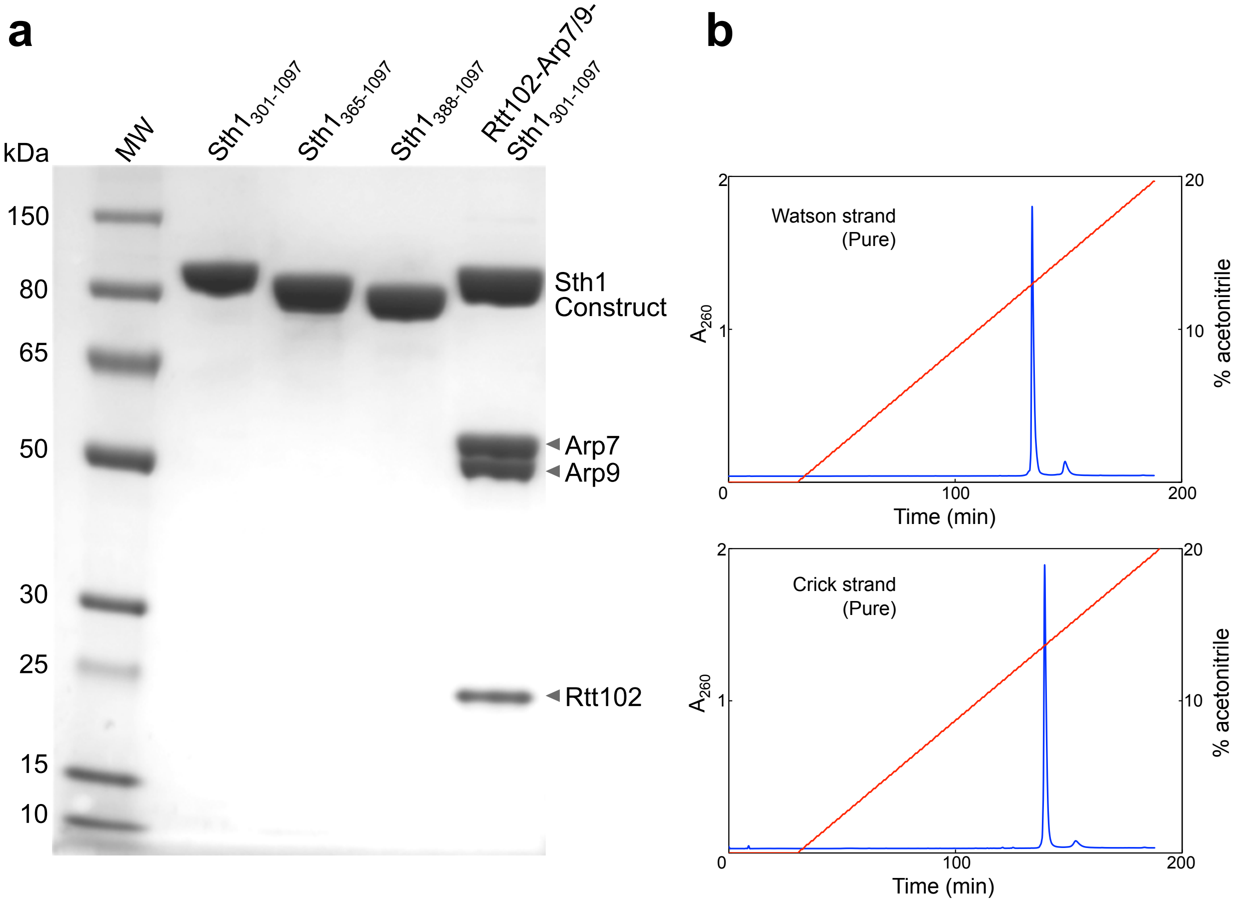

Supplementary Figure 4: Purified protein and DNA samples used in DNA binding studies. (**a**) SDS-PAGE (4-12% gradient) analysis of Sth1 constructs and complexes. (**b**) Reverse-phase elution profiles of the complementary strands of the 20-bp, fluorescin-labeled DNA duplex used in the fluorescence anisotropy experiments described in Fig. 4.

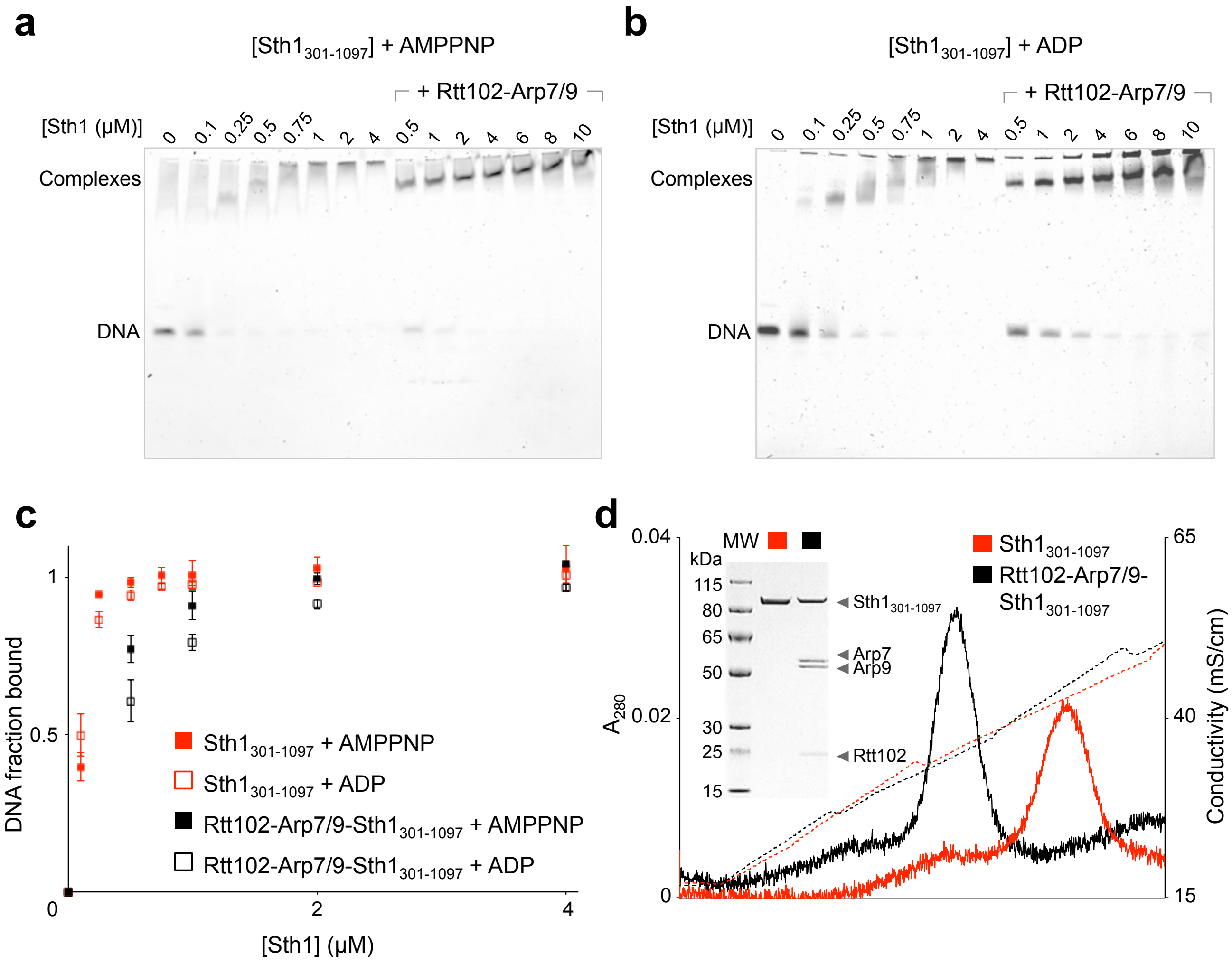

Supplementary Figure 5: Rtt102-Arp7/9 regulates DNA-binding to Sth1. (**a**,**b**) Native PAGE (4-20% gradient) analysis of DNA binding to increasing concentrations of $Sth1_{301-1097}$ or Rtt102-Arp7/9-$Sth1_{301-1097}$. The experiments were performed in the presence of 1 mM AMPPNP (**a**) or 1 mM ADP (**b**). Error bars represent the standard deviation of the mean from three technical replicates of each experiment. (**c**) Quantification of the gel-shift data shown in parts **a** and **b** to a single-site hyperbolic isotherm. The error bars represent the standard deviation of the mean from three gels. (**d**) Elution profiles of $Sth1_{301-1097}$ and Rtt102-Arp7/9-$Sth1_{301-1097}$ on a heparin column. For each sample, ~1 mg of protein was loaded onto the column in 20 mM HEPES pH 7.5, 200 mM NaCl, 5% glycerol, 2 mM DTT, and eluted with a 200–800 mM NaCl gradient. The eluted samples were analyzed by SDS-PAGE (inset).
